# Make Kids Stroke-Smart: A Community Based Interventional Study

**DOI:** 10.7759/cureus.11884

**Published:** 2020-12-03

**Authors:** Dipali Nemade, Mitzi Beckett, Justin Nolte, Vikram Shivkumar

**Affiliations:** 1 Neurology, Marshall University School of Medicine, Huntington, USA; 2 Neurology, Cabell Huntington Hospital, Huntington, USA

**Keywords:** stroke, education, child

## Abstract

Background: Stroke is the third leading cause of death and the major cause of long-term disability in the United States. Timely recognition of symptoms is critical. Family members are crucial in recognizing stroke symptoms since <5% patients can call Emergency Medical Services themselves due to inability to speak or dial the phone. This might be of significance, especially, in family units where children have frequent contact with multiple generations. This study was undertaken to assess and improve the knowledge about stroke amongst children.

Methods: A community-based interventional study was conducted among 305 kids ranging from second to eighth grade. A pre-test questionnaire was administered and later health education regarding stroke was imparted using audiovisual aids. Post-test was done to assess the impact of stroke education. Components of education included were: 1) What is stroke? 2) FAST mnemonic. 3) Time sensitive treatment. 4) Risk factors for stroke 5) How can they help? 6) Whom to call and where to go? The data was compiled and analyzed using Chi square test.

Results: There was a significant lack of knowledge in the pretest groups. The post-test showed statistically significant improvement in all the tested components irrespective of age or grade (p<0.001).

Conclusion: Targeting the younger generation for stroke education is one way to improve community knowledge of stroke symptoms thus increasing the chances that the stroke patient may receive acute stroke therapy. Children can also be used as a conduit to transmit educational information to parents and other family members thus further raising awareness.

## Introduction

Stroke affects more than 795,000 Americans each year and is one of the leading causes of death [1]. Ischemic strokes account for a majority of strokes. Intravenous recombinant tissue plasminogen activator (rtPA) is the treatment of choice for ischemic strokes which present within 4.5 hours [2,3]. Patients who receive rt-PA have a 30% chance of minimal to no disability compared to those who are untreated [4]. Only 4% to 7% of patients with ischemic stroke receive rt-PA in the United States [5]. This has been linked to delay in recognition of symptoms. Stroke patients often do not call emergency services themselves due to impairment of language, motor function, or cognition [6]. Family members, therefore, play a critical role in early diagnosis. Children are increasingly living with grandparents and older parents and can be taught the FAST mnemonic, which stands for F: Facial numbness or weakness, especially on one side, A: Arm numbness or weakness, especially on one side, S: Slurred speech or difficulty speaking, T: Time to call 911. We undertook this study to assess and improve the knowledge about stroke amongst children.

## Materials and methods

A community-based, interventional study was conducted among 305 kids ranging from second to eighth grade at the 11th annual “Brain Expo” held at Marshall University in April 2019. Informed consent was obtained from the parents and the school. The study was approved by the hospital Institutional Review Board. A pre-designed, pre-tested, structured questionnaire in English was administered to the students to assess their existing level of knowledge regarding stroke in the morning session. The components of the questionnaire were: 1) What is stroke? 2) FAST mnemonic. 3) Need for time-sensitive treatment. 4) Risk factors for stroke 5) How can they help? 6) Whom to call and where to go?

Subsequently, health education regarding stroke was imparted to the students through lectures with the help of audio-visual aids. We used models of the brain and cerebral vessels for this purpose. We also had an interactive model that depicted the formation of thrombi and the breakdown of the thrombus using rt-PA. This was followed by a Q&A session to clarify any doubts. In the afternoon session, the same questionnaire was again administered to the students (post-test) to assess the impact of health education. The data obtained was compiled and analyzed statistically using Chi-square test.

## Results

A total of 305 kids participated in the study. There were nine kids in second grade, 87 in third grade, 89 in fourth grade, 92 in fifth grade, 21 in sixth grade, and seven in eighth grade. There was a significant lack of knowledge in the pretest groups. Only a few kids were able to answer the questions correctly. The post-test showed statistically significant improvement in all the tested components irrespective of age or grade (p<0.001) (Table 1 and Table 2).

**Table 1:** Pretest and post-test questions (N=305) FAST= F: Facial numbness or weakness, especially on one side, A: Arm numbness or weakness, especially on one side, S: Slurred speech or difficulty speaking, T: Time to call 911

	Yes	No	Chi square (X^2)^ andpvalues
1. Do you know what stroke is?			
PRE-TEST	17	288	X^2^= 503.336, p<0.001
POST-TEST	294	11	
2. What does the mnemonic FAST mean?			
PRE-TEST	9	296	X^2^= 532.646, p<0.001
POST-TEST	294	11	
3. Do you know treatment of stroke in time sensitive?			
PRE-TEST	8	297	X^2^=543.90, p<0.001
POST-TEST	296	9	
4. Do you know, you can help them reach on time?			
PRE-TEST	19	286	X^2^=499.95, p<0.001
POST-TEST	295	10	
5. Do you know who to call?			
PRE-TEST	51	254	X^2^=404.874, p<0.001
POST-TEST	297	8	
6. Do you know where to take them?			
PRE-TEST	31	274	X^2^= 470.38, p<0.001
POST-TEST	298	7	
7. Do you know the risk factors for stroke?			
PRE-TEST	18	287	X^2^= 499.77, p<0.001
POST-TEST	294	11	

**Table 2:** Chi square (X2) and p values sorted by grade

	2nd grade (N=9)	3rd grade (N= 87)	4th grade (N= 89)	5th grade (N=92)	6th grade (N= 21)	8th grade (N= 7)
What is stroke?	X^2^= 9, p=0.003	X^2^=158.70, p=0.000	X^2^=151.27, p=0.000	X^2^= 150.63, p=0.000	X^2^= 27.77, p=0.000	X^2^= 10.5, p=0.001
FAST mnemonic	X^2^= 9, p=0.003	X^2^=147.30, p=0.000	X^2^=162.36, p=0.000	X^2^= 164.71, p=0.000	X^2^= 42, p=0.000	X^2^= 10.5, p=0.001
Time sensitivity	X^2^=11.45 p=0.001	X^2^=158.70, p=0.000	X^2^=158.58, p=0.000	X^2^= 164.71, p=0.000	X^2^= 42, p=0.000	X^2^= 10.5, p=0.001
You can help	X^2^= 9, p=0.003	X^2^=143.43, p=0.000	X^2^=140.88, p=0.000	X^2^= 172.21, p=0.000	X^2^= 28.56, p=0.000	X^2^= 10.5, p=0.001
Whom to call	X^2^= 18, p=0.000	X^2^=132.94, p=0.000	X^2^=107.84, p=0.000	X^2^= 122.75, p=0.000	X^2^= 17.52, p=0.000	X^2^= 10.5, p=0.001
Where to go?	X^2^= 18, p=0.000	X^2^=143.47, p=0.000	X^2^=124.96, p=0.000	X^2^= 157.53, p=0.000	X^2^= 21, p=0.000	X^2^= 10.5, p=0.001
Stroke risk factors	X^2^= 9, p=0.003	X^2^=132.94, p=0.000	X^2^=162.36, p=0.000	X^2^= 150.63, p=0.000	X^2^= 38.18, p=0.000	X^2^= 10.5, p=0.001

## Discussion

Delay in presentation to the hospital is a major cause for lack of rt-PA use in ischemic strokes [7]. Only 25% of patients reach the hospital within three hours [8]. There is a significant lack of knowledge of stroke symptoms amongst patients and their family members which contributes to the delayed presentation. It is important to address these knowledge gaps to improve patient outcomes. Education programs targeting the individuals at risk can be ineffective because it has been shown that only ~4% patients are likely to call Emergency Medical Systems (EMS) themselves [6]. Therefore, family members, including children can play a critical role in timely treatment.

In our study, the children demonstrated a significant improvement in knowledge of stroke after education. The children were able to recollect common symptoms of strokes, the need for urgent medical attention and the best course of action in such a scenario. This was noted across all age groups, including the youngest children who were in second and third grade. This was consistent with other studies which have shown that children can be trained to identify stroke symptoms and act promptly [9,10].

A limitation of our study was that we did not assess if the children eventually called the Emergency Medical Services (EMS) for real-life stroke occurrences in family members. However, in the following year, we have treated a few stroke patients who were brought in because a child activated the EMS after correctly identifying the symptoms of a stroke. We do not know if these children were part of our study. Other studies have also reported activation of EMS by few children who underwent such education [11,12]. Children have also been shown to serve as an effective conduit for delivery of information in other stroke studies [13-15]. This transfer of information from children to parents has also been documented with other medical conditions. Children are increasingly being raised by grandparents and older parents [16]. Therefore, in addition to transmitting information to adults, children might also be the sole witnesses during a stroke. 

## Conclusions

Time is of the essence in the management of acute ischemic strokes. Delay in presentation to the hospital is a major cause for the lack of rt-PA use in ischemic strokes. Children as young as those in second grade can be educated about stroke. Targeting the younger generation for stroke education is one way to improve community knowledge of stroke symptoms thus increasing the chances that stroke patients may receive timely acute stroke therapy.
